# Safety and Immunogenicity of ChAd63 and MVA ME-TRAP in West African Children and Infants

**DOI:** 10.1038/mt.2016.83

**Published:** 2016-06-28

**Authors:** Muhammed O Afolabi, Alfred B Tiono, Uche J Adetifa, Jean Baptiste Yaro, Abdoulie Drammeh, Issa Nébié, Carly Bliss, Susanne H Hodgson, Nicholas A Anagnostou, Guillaume S Sanou, Ya Jankey Jagne, Oumarou Ouedraogo, Casimir Tamara, Nicolas Ouedraogo, Mirielle Ouedraogo, Jainaba Njie-Jobe, Amidou Diarra, Christopher JA Duncan, Riccardo Cortese, Alfredo Nicosia, Rachel Roberts, Nicola K Viebig, Odile Leroy, Alison M Lawrie, Katie L Flanagan, Beate Kampman, Philip Bejon, Egeruan B Imoukhuede, Katie J Ewer, Adrian VS Hill, Kalifa Bojang, Sodiomon B Sirima

**Affiliations:** 1 Medical Research Council Unit, Fajara, The Gambia; 2 Centre National de Recherche et de Formation sur le Paludisme, Ouagadougou, Burkina Faso; 3 The Jenner Institute Laboratories, University of Oxford, Oxford, UK; 4 Centre for Clinical Vaccinology and Tropical Medicine, The Jenner Institute, Churchill Hospital, Oxford, UK; 5 Keires AG, Bäumleingasse, Basel, Switzerland; 6 ReiThera, Rome, Italy; 7 CEINGE, Naples, Italy; 8 Department of Molecular Medicine and Medical Biotechnology, University of Naples Federico II, Naples, Italy; 9 European Vaccine Initiative, UniversitätsKlinikum Heidelberg, Heidelberg, Germany; 10 Current address: Department of Immunology, Monash University, Prahran, Melbourne, Australia; 11 Kenya Medical Research Institute, Centre for Geographical Medical Research (Coast), Kilifi, Kenya

## Abstract

Malaria remains a significant global health burden and a vaccine would make a substantial contribution to malaria control. Chimpanzee Adenovirus 63 Modified Vaccinia Ankara Multiple epitope thrombospondin adhesion protein (ME-TRAP) and vaccination has shown significant efficacy against malaria sporozoite challenge in malaria-naive European volunteers and against malaria infection in Kenyan adults. Infants are the target age group for malaria vaccination; however, no studies have yet assessed T-cell responses in children and infants. We enrolled 138 Gambian and Burkinabe children in four different age-groups: 2–6 years old in The Gambia; 5–17 months old in Burkina Faso; 5–12 months old, and also 10 weeks old, in The Gambia; and evaluated the safety and immunogenicity of Chimpanzee Adenovirus 63 Modified Vaccinia Ankara ME-TRAP heterologous prime-boost immunization. The vaccines were well tolerated in all age groups with no vaccine-related serious adverse events. T-cell responses to vaccination peaked 7 days after boosting with Modified Vaccinia Ankara, with T-cell responses highest in 10 week-old infants. Heterologous prime-boost immunization with Chimpanzee Adenovirus 63 and Modified Vaccinia Ankara ME-TRAP was well tolerated in infants and children, inducing strong T-cell responses. We identify an approach that induces potent T-cell responses in infants, which may be useful for preventing other infectious diseases requiring cellular immunity.

## Introduction

Malaria remains a significant public health problem, especially among under 5 years old children in Africa. Although widespread malaria control interventions have led to a remarkable decline in malaria associated deaths; there has been an increasing concern about emerging threats of resistance to artemisinin-based antimalarial drugs and insecticide-treated nets.^1^ This concern has rekindled the need for additional strategies in reducing the burden of malaria. One study also suggested that malaria associated death could be twice more than that was previously reported; with most deaths occurring in African children.^2^ An effective malaria vaccine has therefore been agreed to be a valuable complementary tool to optimize existing malaria control strategies and contribute to elimination of malaria in Africa.^3^

Current malaria vaccine candidates are directed against the human and mosquito stages of the parasite life cycle, but so far, the leading candidate RTS,S/AS01 has demonstrated only partial protection among young African children,^4,5^ with suboptimal durability. Most candidate vaccines in clinical development are based on the classical vaccination approach of a single vaccine administered in a homologous prime–boost schedule which induce primarily neutralizing antibodies but weak CD4^+^ and no CD8^+^ T-cells.^6^ This approach may account for the inadequate protection generated by the candidate vaccines, and justifies the need for other approaches to induce and sustain strong T-cell responses and help alleviate the huge mortality associated with malaria. Hence, the innovative approach of combining different vaccine modalities to complement and induce broad and sustainable immunity was developed.^6^

Heterologous prime-boost regimens with adenovirus priming and MVA boosting are currently being developed for a wide range of diseases including respiratory syncytial virus, malaria, tuberculosis, human immunodeficiency virus, pandemic influenza, hepatitis C, Ebola virus disease, and cancer.^7,8,9,10,11,12,13^ We report here the first evaluation of this approach to inducing T-cell and antibody responses in young children and infants. This is also to our knowledge, the first evaluation of a simian adenovirus in children or infants. Using the Chimpanzee Adenovirus 63 (ChAd63) and Modified Vaccinia Ankara ME-TRAP (MVA ME-TRAP) vectors, we undertook phase 1 dose-escalation and age de-escalation studies to assess safety and immunogenicity in malaria-exposed children and infants.

Safety of human recombinant adenovirus vaccines has been assessed previously in infants aged 6–9 months that were immunized with AERAS-402, a novel TB vaccine expressing three antigens from *Mycobacterium tuberculosis*^14^ and an MVA encoding the antigen85A, MVA85A, has been assessed as a single dose vectored vaccine in African infants in phase 1 and 2a trials.^15,16^ Here we describe the safety of a recombinant chimpanzee adenovirus in a prime-boost regimen with MVA boosting in younger infants aged 10 weeks at first immunization that would be the preferred target age for a malaria vaccine.

Two clinical trials were performed in Sukuta in the western region of The Gambia where malaria transmission has declined substantially since 2003, although modest levels of transmission occur following seasonal rains.^17^ The third study was undertaken in the Cascades region of south western Burkina Faso, where transmission is again highly seasonal, but with a much higher incidence of malaria than in The Gambia, with an average of two confirmed episodes per child per year.^18^ Both sites represent settings whereas an effective malaria vaccine might be deployed usefully and we describe here safety profiles and preliminary T-cell responses from four groups of children of decreasing ages from 6 years to 10 weeks old.

## Results

### Safety and reactogenicity

In group 1 involving Gambian children aged 2–6 years, all adverse events (AEs) reported after vaccination with either high or low dose of ChAd63 ME-TRAP and MVA ME-TRAP were mild in intensity, with pain at the injection site (7/24, 29%), and documented fever (3/24, 12.5%) the most frequently observed symptoms (see **Supplementary Tables S1–S6**). The higher dose of ChAd63 produced more local solicited AEs than the lower dose. Among participants in group 2, Gambian infants aged 5–12 months, all AEs reported after vaccination with either dose of ChAd63 ME-TRAP were mild and resolved within 1 day of onset, with fever the most commonly reported symptom (4/24, 17%, among vaccinees versus 2/24, 8% in unvaccinated controls).

Among group 3 participants, Gambian infants aged 10 weeks at first vaccination; all AEs related to ChAd63 ME-TRAP were mild and resolved within 1 day of onset. There were no unsolicited AEs related to ChAd63, however a single serious adverse event (SAE) was recorded due to hospital admission for gastroenteritis on day 1, postvaccination that was considered unlikely to be related to vaccination. After administration of MVA ME-TRAP, eight AEs possibly related to vaccination occurred with fever the most commonly reported symptom (6/12, 50% of vaccinated infants, compared with 1/12, 8% unvaccinated controls). All were mild in intensity and all hematological and biochemical tests were within normal ranges. No child in groups 1–3 developed clinical malaria during the study. Among the 30 Burkinabe infants and children aged 5–17 months in group 4, the safety profile was broadly similar to that observed in Gambian vaccine recipients aged 5–12 months (see **Supplementary Tables S1–S6**).

### Immunogenicity

**Figure 1:**
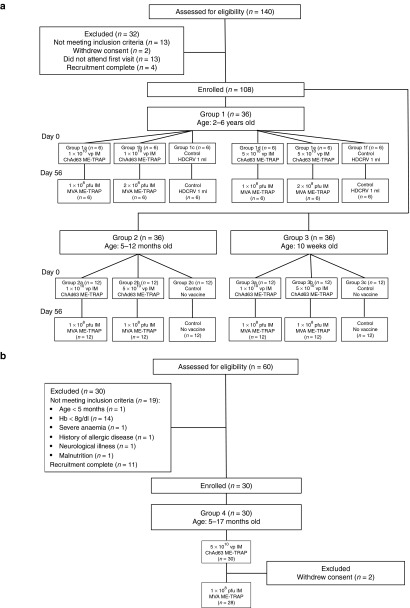
**Participant flow chart for malaria vectored vaccine trials in Gambian and Burkinabe children and infants**. (**a**) CONSORT flow chart for groups 1, 2, and 3 in The Gambia. (**b**) CONSORT flow chart for group 4 in Burkina Faso.

**Figure 2:**
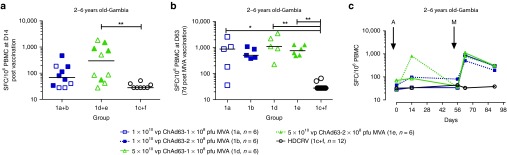
**Immune responses following ChAd63/MVA ME-TRAP vaccinations in Gambian children**. (**a**) Individual responses by dose of ChAd63 ME-TRAP 14 days after vaccination. Increases in response were significant in volunteers receiving 5 × 10^10^ vp i.m. compared with rabies vaccine (Groups 1d and 1e compared with 1c and 1f, *P* = 0.004, Kruskal–Wallis). Lines represent group median. (**b**) Individual responses 7 days after boosting with MVA ME-TRAP (day 63), responses among recipients of the higher dose of MVA (2 × 10^8^ pfu i.m.) increased significantly compared with rabies vaccination. Boosting with the lower dose of MVA (1 × 10^8^ pfu i.m.) also increased responses significantly in group 1a. There was no significant effect of priming or boosting dose on the magnitude of the ELISPOT response at day 63 (1a compared with 1b or 1d compared with 1e, two-tailed Mann–Whitney test). (**c**) Time course of median ELISPOT responses in 2–6 years old children receiving either ChAd63-MVA ME-TRAP or HDCRV. Abbreviations: ChAd63, chimpanzee adenovirus serotype63; ME-TRAP, multiple epitope–thrombospondin-related adhesion protein; MVA, modified vaccinia virus Ankara; pfu, plaque-forming units; vp, viral particles; i.m., intramuscularly; HDCRV, human diploid cell rabies vaccine. All comparisons between time points use Kruskal–Wallis with Dunn's multiple comparison test: *P < 0.05; **P < 0.01, ***P < 0.001.

*Dose-finding in group 1 in The Gambia.* Children aged 2–6 years (group 1) received either high or low dose ChAd63 ME-TRAP boosted with either high or low dose MVA (**Figure 1**) or vaccinated with rabies vaccine as a control. An increase in ME-TRAP-specific IFNγ-secreting T-cells as measured by Enzyme-Linked ImmunoSpot (ELISPOT) assay was apparent after vaccination with ChAd63 ME-TRAP in all children in group 1, aged 2–6 years old (**Figure 2a**,**c**), compared with Human Diploid Cell Rabies Vaccine (HDCRV)-vaccinated controls. For those vaccinated with the higher dose of 5 × 10^10^ viral particles (vp) intramuscularly (i.m.) of ChAd63 ME-TRAP, the increase in ELISPOT response was significant compared with the recipients of rabies vaccine (groups 1d and 1e: 298 spot-forming cells (SFC) per million peripheral blood mononuclear cell (PBMC) with interquartile range (IQR) 53–1040, compared with 1c and 1f: 28 SFC, IQR 28–39 SFC, *P* = 0.004, Kruskal–Wallis Test).

After a booster vaccination with MVA ME-TRAP, IFNγ ELISPOT responses among recipients of the lower dose of MVA (1 × 10^8^ pfu i.m.) increased significantly from baseline (Group 1a preboost: 69 SFC, IQR 33–180, postboost: 882 SFC, IQR 110–1838, *P* < 0.05, Kruskal–Wallis test). Boosting with the higher dose of MVA (2 × 10^8^ plaque-forming units (pfu) i.m.) significantly increased responses compared with rabies vaccination (*P* < 0.01, Kruskal–Wallis test for groups 1b and 1e compared with 1c and 1f, **Figure 2b**). There was no significant effect of priming or boosting dose on the magnitude of the ELISPOT response at day 63 (1a compared with 1b and 1d compared with 1e, two-tailed Mann–Whitney test).

**Figure 3:**
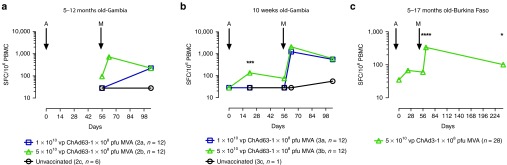
**Immune responses following ChAd63/MVA ME-TRAP vaccinations in Gambian and Burkinabe infants**. (**a**) Time course of median ELISPOT responses in 5–12 months old. Only available data points are shown. (**b**) Time course of median ELISPOT responses in babies aged 10 weeks at first vaccination. Responses were significantly higher at day 21 after ChAd63 ME-TRAP vaccination in the group that received the higher dose (*P* = 0.002, group 3a compared with 3b, two-tailed Mann-Whitney test). (**c**) Time course of median ELISPOT responses among 28 children aged 5–17 months old in Burkina Faso. All received 5 × 10^10^ vp i.m. ChAd63 ME-TRAP followed by 1 × 10^8^ pfu i.m. MVA ME-TRAP 8 weeks later. Responses were significantly higher than preimmunization levels at D63 (*P* < 0.0001) and D243 (*P* < 0.05). Abbreviations: ChAd63, chimpanzee adenovirus serotype63; ME-TRAP, multiple epitope–thrombospondin-related adhesion protein; MVA, modified vaccinia virus Ankara; pfu, plaque-forming units; vp, viral particles; i.m., intramuscularly; HDCRV, human diploid cell rabies vaccine. All comparisons between time points use Kruskal–Wallis with Dunn's multiple comparison test: *P < 0.05; **P < 0.01; **P < 0.001.

*Age de-escalation in groups 2, 3 and 4 in The Gambia and Burkina Faso.* As no effect of dose was apparent in the first group; all children in the subsequent groups received 1 × 10^8^ pfu i.m. of MVA ME-TRAP. Age de-escalation and dose escalation continued for ChAd63 ME-TRAP as adenoviral vectors have undergone less evaluation in young children than MVA. ELISPOT data for the 5–12 months old group was incomplete due to poor lymphocyte viability, although in group 2b that received the higher dose of ChAd63 ME-TRAP the immune response peaked after MVA vaccination at 720 SFC (IQR 288–982 SFC), **Figure 3a**. In infants immunized at 10 weeks of age, there was a significant effect of dose after priming at day 21 after vaccination (*P* = 0.002, two-tailed Mann–Whitney test, **Figure 3b**), but after boosting with MVA there was no significant difference in responses between vaccinated groups (*P* = 0.24). Responses in vaccinated children remained significantly above those in unvaccinated controls 5 weeks after boosting (*P* < 0.05, Kruskal–Wallis with Dunn's post test). In children aged 5–17 months vaccinated in Burkina Faso, responses increased slightly after boosting at day 21 and peaked 7 days after boosting with MVA at 336 SFC (IQR 206–576, *P* < 0.001 compared with prevaccination, Kruskal–Wallis test with Dunn's post test, **Figure 3c**). Responses were still significantly higher than prevaccination, 6 months after the MVA immunization (72 SFC, IQR 35–128, *P* < 0.05, Kruskal–Wallis test with Dunn's post-test).

## Discussion

We report on the first use of a chimpanzee adenovirus vectored vaccine and of chimpanzee adenovirus prime—MVA boost regimens in children and infants, and identify a surprising ability of these vectors to induce higher levels of T-cells compared with responses in adults.

Prime-boost vaccination with ChAd63 and MVA ME-TRAP has demonstrated an acceptable safety profile in four cohorts of children of decreasing ages in The Gambia and Burkina Faso. Vaccination was particularly well tolerated in the group of infants aged 10 weeks at the time of vaccination with ChAd63, where all vaccine-related AEs were mild and resolved within 1 day. In addition, this regimen elicited substantial cellular immunity in the younger age groups, with levels of TRAP-specific T-cells highest in 10-week-old babies.

Previous studies with the same antigen encoded in fowlpox and MVA vectors failed to elicit protection against malaria in Kenyan children aged 1–6 years due to reduced immunogenicity relative to malaria-naive adults, presumably due to malaria-associated immunosuppression.^19,20^ The substitution in this regimen of the heterologous adenovirus for the fowlpox priming vector has increased the T-cell immunogenicity of this antigen 10-fold in the comparable age groups, based on *ex vivo* ELISPOT responses. This demonstrates the potency of the ChAd63 vector in combination with MVA encoding the same antigenic insert. Comparison of this regimen in Gambian and Kenyan adults was fourfold higher than in the fowlpox-MVA regimen, suggesting the potency of the ChAd63 vector may be higher in children than adults. A possible explanation for this is that the malaria-associated immunosuppression has less effect on responses to adenoviral vectors than poxviral vectors, perhaps as a result of different receptor usage or stimulation of alternative pathways of innate immunity. T-cell responses remained above prevaccination thresholds for at least 6 months after boosting demonstrating useful durability.

We report here a detailed analysis of the safety of ChAd63 and MVA ME-TRAP in four groups of African children that would benefit from an effective malaria vaccine. These vaccines show remarkable safety with very acceptable reactogenicity profiles for both vaccines at two dose levels, highlighting the potential utility of these viral vectors for childhood immunization against other infections. We have described in detail the potent cellular immunogenicity of these vaccines in children aged 5–17 months in Burkina Faso and 5–12 months or 10 weeks old in The Gambia. This regimen is known to elicit CD8^+^ T-cell-mediated efficacy against CHMI in malaria-naive adults^9^ and therefore high frequencies of similar T-cell populations in children that are the target for a malaria vaccine is a promising observation for future efficacy.

These data support further evaluation of this regimen in phase 2 studies, particularly in younger age groups of children to assess efficacy against malaria in regions of malaria transmission. Further trials are underway to assess efficacy in semi-immune adults and 5–17 months old infants and children, and to determine schedules for optimal deployment within the World Health Organization expanded program of immunization.

## Materials and Methods

***Objectives.*** The primary objective was to evaluate the safety and reactogenicity of the ChAd63 ME-TRAP and MVA ME-TRAP vaccines in malaria-experienced Gambian and Burkinabe children. Secondary objectives were to evaluate the cellular and humoral immunogenicity of the vaccines in semiimmune Gambian and Burkinabe children in two settings of varying seasonal malaria transmission, while the tertiary objective was to compare the immunogenicity of the low and high doses of MVA ME-TRAP (Gambian children only).

***Study settings.*** The first clinical trial (group 1) took place from December 2010 to December 2011 at the Sukuta field site of the Medical Research Council, The Gambia. Sukuta is a peri-urban village located about 30 km south of the capital Banjul. The Sukuta field site previously served as the base for the phase 1 trials of ChAd63 MVA ME-TRAP vaccine in adults (18 years). In this region, malaria transmission is highly seasonal, occurring almost exclusively during the rainy season (July to December) with greatest incidence in September to November. *Anopheles gambiae* is the principal malaria vector. Previous studies have documented a decline in incidence of malaria in The Gambia^17,21^; however, an upsurge in malaria cases has been reported in different parts of the country.^22^ The second clinical trial (groups 2 and 3) took place in the same setting between September 2011 and March 2013.

The third clinical trial (group 4) took place from December 2012 to September 2013 in Banfora Health District in the Cascades region of South Western Burkina Faso, about 400 km south-west from the capital Ouagadougou. Malaria transmission is stable during the year, with increased levels during the rainy season from May to November, peaking from May to September.^18^ *A. gambiae* is the principal malaria vector.

***Participants.*** After local community meetings held by the trial teams in both sites, parents of potential participants were invited to the trial site for eligibility screening tests. Before enrolment, mothers gave a detailed medical history for the child. The child then underwent physical examination and laboratory evaluation of blood samples to determine suitability for enrolment. The children were eligible for enrolment if they were within the correct age group for the relevant study, in good health, a parent or guardian provided informed consent for participation in the study and residence in the study area was anticipated during the vaccination and follow-up period. Exclusion criteria included any evidence of chronic illness or of hematological, renal or hepatic pathology: hemoglobin level <8 g/dl for children under 2 years or 9 g/dl for older children; severe malnutrition; positive malaria antigen test (Gambian trials only); positive HIV serology test for children above 2 years or positive maternal HIV test for younger children; clinically significant serum biochemistry results; prior receipt of an investigational malaria vaccine; recent or planned use of any investigational drug, vaccine, immunoglobulin or any blood product; use of immunosuppressant drugs; confirmed or suspected immunodeficiency; history of surgical splenectomy; concurrent participation in another clinical trial.

***Study design.*** We conducted three phase 1b studies. The first study (group 1, aged 2–6 years) in The Gambia was the pediatric arm of a phase 1b single-blind, randomized controlled, dose-escalation study in adults that has been reported previously.^23,24^ The second study, also in The Gambia, was a subsequent single-blind randomized controled, dose-escalation study in children aged 5–12 months (group 2) and 10 weeks (group 3) at vaccination with ChAd63 ME-TRAP. The third study was a phase 1 open-label safety lead-in group of a larger phase 2b study in Burkina Faso in children aged 5–17 months at first vaccination (group 4). Protocols and CONSORT checklists are provided in Supplementary Information Protocol S1 (group 1, The Gambia), Protocol S2 (groups 2 and 3, The Gambia), Protocol S3 (group 4, Burkina Faso), and Checklists S1–S3. All vaccinations were intramuscular with group 1 receiving doses in the deltoid region of the arm, while all other groups were vaccinated in the anterolateral thigh. A control group was added to group 1 because of the anticipated high frequency of concurrent diseases in the study age group of 2–6 years and also to aid objective assessment of the relationship of AEs to vaccination.

***Recruitment.*** Two hundred children were screened for eligibility across the three trials and 138 eligible children were enrolled, vaccinated, and followed up (**Figure 1**). Two children from group 1 and two children from group 4 withdrew before follow up was completed. The parents of one study child in group 1, relocated outside the study area while the mother of another child withdrew consent before MVA boost vaccination. Similarly in group 4, mothers of two children withdrew consent before MVA boost vaccination. Trial groups are shown in **Table 1**. Baseline demographic data for each group are shown in **Table 2**.

***Sample size.*** These phase 1b trials were not powered to detect differences between groups. The sample size was based on general acceptance of this size for initial assessment of safety, tolerability and immunogenicity of the investigational vaccines in a malaria endemic area and this size balances the need to avoid exposing a large group of study participants to an unknown risk with the need for useful safety and immunogenicity data from an adequate sample size.

***Interventions.*** The Clinical Biomanufacturing Facility (CBF), University of Oxford, UK and IDT, Germany manufactured ChAd63 ME-TRAP and MVA ME-TRAP under Good Manufacturing Practice conditions, respectively as previously described.^9^

Human Diploid Cell Rabies Vaccine (HDCRV, Sanofi Pasteur MSD, Mallards Reach, Berkshire, UK) was chosen as the comparator vaccine because rabies is endemic in The Gambia and antirabies vaccines were not readily accessible for preexposure prophylaxis; hence the investigators decided that giving HDCRV might benefit the study children. In groups 2 and 3, notreatment controls were included, and there was also no control group in group 4 as the subsequent larger phase 2 study had a rabies vaccine control arm.

***Randomization in groups 1, 2 and 3, and blinding.*** An independent statistician at the Centre for Statistics in Medicine, Oxford performed a stratified randomization of participants (stratified by age into two categories and split by the median values of ages of children recruited). The list of eligible children after screening was sent to the statistician who carried out the randomization. The statistician had no knowledge of the participants, except the age, as this was required for the stratification. The children were randomly allocated to six groups in dose-escalated fashion. This was done to determine the tolerable doses as this was the first time ChAd63 and MVA.ME-TRAP vaccines were being administered to Gambian children. The investigators and the vaccinators were unblinded to the group allocations. However, the study children's parents/carers and field workers who conducted postvaccination assessment of reactogenicity and solicited symptoms were blinded to the group allocations.

For group 1, 36 eligible children were randomized to receive either group 1a: low dose ChAd63 ME-TRAP (1 × 10^10^ vp) followed by low dose MVA ME-TRAP (1 × 10^8^ pfu); group 1b: low dose ChAd63 ME-TRAP (1 × 10^10^ vp) followed by high dose MVA ME-TRAP (2 × 10^8^ pfu); group 1c: Control HDCRV 1 ml followed by HDCRV 1 ml. Group 1d: high dose ChAd63 ME-TRAP (5 × 10^10^ vp) followed by low dose MVA ME-TRAP (1 × 10^8^ pfu); group 1e: high dose ChAd63 ME-TRAP (5 × 10^10^vp) followed by high dose MVA ME-TRAP (2 × 10^8^ pfu); group 1f: Control HDCRV 1 ml followed by HDCRV 1 ml i.m. All vaccinations were separated by an eight-week interval.

For groups 2 and 3, 36 eligible children in each group were randomized to receive either group a: low dose ChAd63 ME-TRAP (1 × 10^10^vp) followed by low dose MVA ME-TRAP (1 × 10^8^ pfu); group b: low dose ChAd63 ME-TRAP (1 × 10^10^ vp) followed by high dose MVA ME-TRAP (2 × 10^8^ pfu); group c: no vaccine. For group 4, all 30 children received high dose ChAd63 ME-TRAP (5 × 10^10^ vp) followed by low dose MVA ME-TRAP (1 × 10^8^ pfu).

Administration of ChAd63 ME-TRAP and MVA ME-TRAP occurred in three escalated stages. Each group was age stratified to ensure that any imbalance in safety and reactogenicity rates was not due to a disproportion of young children in any one cohort. Vaccinations of study children with ChAd63 ME-TRAP were staggered from each other by 2 weeks and by 1 week in MVA-ME-TRAP vaccinations. A safety report was produced prior to each dose escalation and safety assessment approval by Local Safety Monitor (LSM) and Data Safety Monitoring Board (DSMB) was achieved before proceeding to the next stage. The LSM and DSMB also reviewed all AEs occurring in the 14 days immediately following any vaccination that preceded a dose escalation. Written approval from the DSMB and concurrence by the LSM were required prior to any subsequent dose escalation.

***Outcomes.*** The primary endpoint was safety measured as (i) occurrence of solicited symptoms during a 3-day follow-up period after each immunization; (ii) occurrence of unsolicited symptoms during a 28-day follow-up after each vaccination; (iii) occurrence of abnormal laboratory results during study period; and (iv) occurrence of SAEs during the study period. The secondary outcomes were T-cell responses as determined by ELISPOT and flow cytometry with intracellular cytokine staining and anti-TRAP antibody titers. Time points for assessment of immunogenicity varied by trial according to logistical, ethical, and clinical considerations. For group 1, samples were collected on study days 0, 14, 56, 63, 90, and 300; for groups 2 and 3, samples were collected on days 0, 21, 56, 63, and 105; for group 4, samples were collected on days 0, 21, 56, 63, and 243.

***Assessment of primary endpoints (safety and reactogenicity).*** Adverse events were graded by intensity and judged for relatedness to study vaccines. Mild AEs were easily tolerated, causing minimal discomfort. Moderate AEs were sufficiently discomforting to interfere with normal activities. Severe adverse events prevented normal daily activities. Swelling, redness, and fever had specific definitions not based on interference with daily activities. Injection site swelling and redness were graded based on their widest dimension: mild, 0–20 mm; moderate, 20–50 mm; and severe >50 mm. Fever was classified as severe if the axillary temperature was ≥40°C. For laboratory tests, toxicity grading was adapted to normal reference ranges determined for the local pediatric population.

Following each vaccination, all study children were directly observed in the clinic for 1 hour; followed up for occurrence of solicited symptoms for three consecutive days; unsolicited symptoms for 30 days, and laboratory abnormalities and SAEs for the entire study period. Trained field workers visited the children at home daily for the 3 days after each vaccination to administer a reactogenicity questionnaire to the parents/guardians that included history of fever, vomiting, diarrhea, reduced oral intake, and reduced activities. The field worker also examined the child for expected local AEs (swelling, tenderness, limitation of arm movement, redness, and desquamation at the site of injection) and fever. Pain at the injection site was graded on a scale of 0–3 (where 0 = no pain, 1 = painful to touch, 2 = pain when arm is touched, and 3 = severe pain at rest).

The study participants were subsequently evaluated at the clinic on study days 14, 63, 90, and 300. Clinical evaluations consisted of measurement of vital signs and assessment for local injection site, general solicited symptoms and signs. Local solicited symptoms and signs included pain, swelling, and redness at injection site, while systemic solicited symptoms and signs included fever (axillary temperature >38°C), reduced oral intake, reduced activity, and vomiting. Any other symptoms or signs were considered to be unsolicited. Solicited symptoms were considered to be related to the study vaccines. Unsolicited symptoms and signs were recorded during the 30 days after each vaccination while SAEs were monitored throughout the study period. Blood samples were collected at screening, on vaccination days and study days 14, 63, 90, and 300 to determine complete blood count, alanine aminotransferase, and serum creatinine.

***Blood processing.*** Blood samples were stored at room temperature prior to processing, which was completed within 6 hours of venepuncture. PBMC were separated by density gradient centrifugation from heparinized whole blood and resuspended in Roswell Park Memorial Institute medium containing 10% heat-inactivated, batch-tested, and sterile-filtered fetal bovine serum (Labtech International), 1% L-glutamine, and 1% penicillin/streptomycin. Cell counts were performed using Trypan blue staining and a microscope according to an established standard operating procedure in the lab. Blood processing was harmonized between the labs in The Gambia and Burkina Faso. ELISPOT assays performed in the adult trial were performed in the same laboratory and were harmonized with procedures in these studies.

***Ex vivo ELISPOT assays.*** *Ex vivo* (18 hours stimulation) ELISPOT assays were performed using Multiscreen IP ELISPOT plates (Millipore, Watford, UK), human IFNγ SA-ALP antibody kits (Mabtech, Nacka, Sweden) and BCIP NBT-plus chromogenic substrate (Moss, Pasadena, MD). Cells were cultured in Roswell Park Memorial Institute medium (Sigma, St.Louis, MO) containing 10% heat-inactivated, sterile-filtered fetal calf serum, previously screened for low reactivity (Labtech International, East Sussex, UK), supplemented with 1% L-glutamine and 1% penicillin/streptomycin. Antigens were tested in duplicate with either 200,000 or 250,000 PBMC added to each well of the ELISPOT plate. TRAP peptides were 20 amino acids in length, overlapping by 10 amino acids (NeoBioLab, London, UK), assayed in six pools of 7–10 peptides at 10 μg/ml. Responses were averaged across duplicates, responses in unstimulated (negative control) wells were subtracted and then responses in individual pools were summed for each strain of the TRAP antigen Staphylococcal enterotoxin B (0.02 μg/ml) and phytohemmagglutinin-L (10 μg/ml) were used as a positive control. Plates were counted using an AID automated ELISPOT counter (AID Diagnostika GmbH, Strassberg, Germany algorithm C), using identical settings for all plates, and counts were adjusted only to remove artifacts. Responses to the negative control were always <154 SFC per million PBMC and the median across all trials was 12 SFC per million PBMC. Pools were considered positive if the response was >12 SFC per million PBMC and two times higher than the negative control for that assay. The lower limit of detection for the assay was 28 SFC for ME-TRAP.

***Statistical methods.*** Data entry was double-entered on OpenClinica^®^ software and analyses performed using STATA Release statistical software version 11.1 (StataCorp LP, College Station, TX). For categorical variables, data were summarized using numbers and percentages. The incidence of solicited and unsolicited adverse events was compared between the comparator and test vaccine groups using Fisher's exact test. For continuous variables, the median, and interquartile range or geometric means with 95% confidence intervals (CI) were used to summarize the data. Participants were analyzed according to the treatments they received.

Group data are geometric means unless otherwise stated with 95% CI. The matched pair analysis excludes volunteers with missing data at any time point. A Kruskal–Wallis test was used to compare increases in T-cell frequencies in time courses with Dunn's multiple comparisons post-test used to compare response pre and postvaccination. For statistical analyses, an alpha-level of 0.05 was considered significant and all *P*-values are two-tailed. All analyses were performed in GraphPad Prism, Mac version 6. (GraphPad Software, La Jolla CA).

***Ethics and regulatory approval.*** An independent DSMB was appointed before the trials began to provide oversight and review the safety data reports as the trials progressed. Experienced local pediatricians served as LSM and, along with the DSMB, reviewed all safety data between dose escalations. In addition, trials were conducted according to ICH Good Clinical Practice guidelines and were monitored by an external organization (Appledown Clinical Research., Gt Missenden, Bucks, UK). The Gambian Government/Medical Research Council Joint Ethics Committee, The Gambia Medicines Board, the Burkina Faso Ministry of Health and Institutional Bioethics Committee, the UK Medicines and Healthcare products Regulatory Authority and Oxford Tropical Research Ethics Committee (OXTREC Numbers: 64-09, 26-11, 41-12) granted approval of the study protocol. All three trials were registered with http://www.clinicaltrials.gov/.

**SUPPLEMENTARY MATERIAL** **Table S1.** Local solicited adverse events during 3 day follow up after ChAd63 vaccination (or first dose HDRCV where relevant). **Table S2.** Systemic solicited adverse events during 3 day follow up after ChAd63 vaccination (or first dose HDRCV where relevant). **Table S3.** Local solicited adverse events during 3 day follow up after MVA vaccination (or second dose HDRCV where relevant). **Table S4.** Systemic solicited adverse events during 3 day follow up after MVA vaccination (or second dose HDRCV where relevant). **Table S5.** Incidence of unsolicited AEs in 28 days post immunisation with ChAd63 ME-TRAP. **Table S6.** Incidence of unsolicited AEs in 28 days post immunisation with MVA ME-TRAP. **Supplementary Information**

## Author Contributions

M.O.A. , A.B.T., K.B., and S.B.S. contributed equally to this work.

## Trial Registration

All three trials were registered with clinicaltrials.gov (NCT01373879, NCT01450293, NCT01635647) and the Pan African Clinical Trials Registry, www.pactr.org, (PACTR201204000362870, PACTR201401000363170, PACTR201208000404131).

## Figures and Tables

**Table 1:**
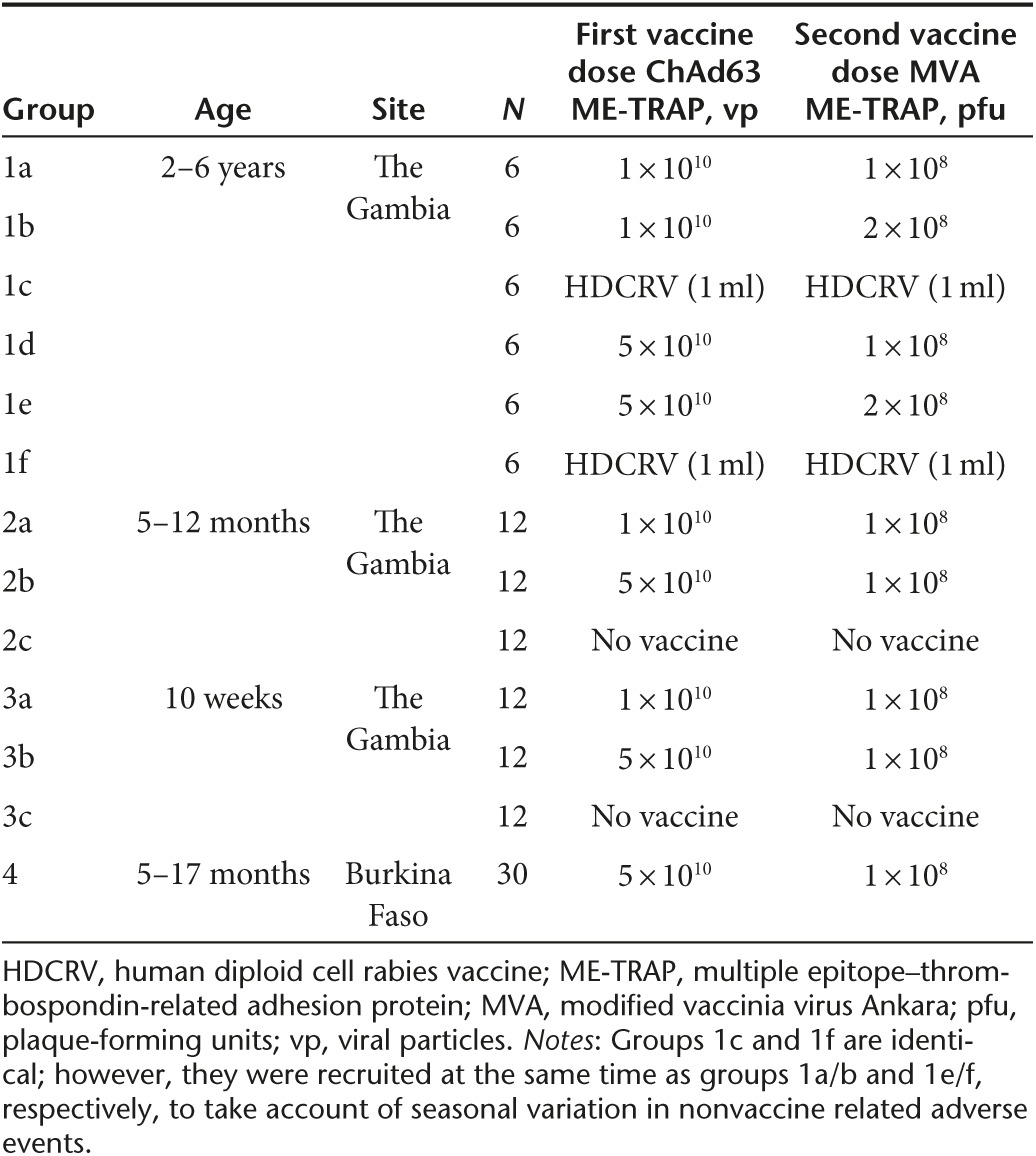
Study outline

**Table 2:**
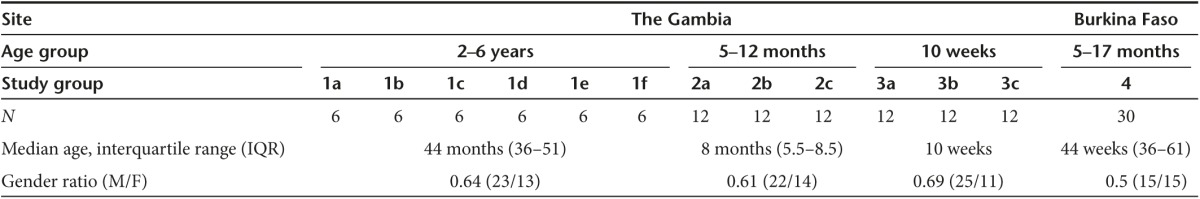
Baseline demographics of enrolled trial participants
