# Supplementary material for: Safety and Immunogenicity of ChAd63 and MVA ME-TRAP in West African Children and Infants
Source: Mol Ther. 2016 Jun 28;24(8):1470–7. doi: 10.1038/mt.2016.83 (PMC5010143; doi:10.1038/mt.2016.83)
Supplement: Supplementary Information [file mt201683x1.doc]

**Safety and Immunogenicity of ChAd63 and MVA ME-TRAP in**

**West African children and infants**

Muhammed O. Afolabi^1*^, Alfred B. Tiono^2*^, Uche J. Adetifa^1^, Jean Baptiste Yaro^2^, Abdoulie Drammeh^1^, Issa Nébié^2^, Carly Bliss^3^, Susanne H. Hodgson^4^, Nicholas A. Anagnostou^4^, Guillaume S. Sanou^2^, Ya Jankey Jagne^1^, Oumarou Ouedraogo^2^, Casimir Tamara^2^, Nicolas Ouedraogo^2^, Mirielle Ouedraogo^2^, Jainaba Njie-Jobe^1^, Amidou Diarra^2^, Christopher J. A. Duncan^4^, Riccardo Cortese ^5^, Alfredo Nicosia ^6,7,8^, Rachel Roberts^4^, Nicola K. Viebig^9^, Odile Leroy^9^, Alison M. Lawrie^4^, Katie L. Flanagan^1$^, Beate Kampman^1^, Philip Bejon^10^, Egeruan B. Imoukhuede^4^, Katie J. Ewer^3^, Adrian V. S. Hill^3,4^, Kalifa Bojang^1*^, Sodiomon B Sirima^2*^

**Affiliations**

^1^ Medical Research Council Unit, Fajara, The Gambia.

^2^ Centre National de Recherche et de Formation sur le Paludisme, Ouagadougou, Burkina Faso

^3^ The Jenner Institute Laboratories, University of Oxford, Old Road Campus Research Building, Oxford, UK.

^4^ Centre for Clinical Vaccinology and Tropical Medicine, The Jenner Institute, Churchill Hospital, Oxford, UK.

5 Keires AG, Bäumleingasse 18, CH 4051 Basel, Switzerland

^6^ ReiThera, Viale Città d’Europa 679, 00144, Rome, Italy.

^7^ CEINGE, Via Gaetano Salvatore 486, 80145, Naples, Italy

^8^ Department of Molecular Medicine and Medical Biotechnology, University of Naples Federico II, Via S. Pansini 5, 80131, Naples, Italy

^9^ European Vaccine Initiative, UniversitätsKlinikum Heidelberg, Im Neuenheimer Feld 326 - 3. OG 69120 Heidelberg, Germany

^10^ Kenya Medical Research Institute, Centre for Geographical Medical Research (Coast), Kilifi, Kenya.

^$^ Current affiliation: Dept. of Immunology, Monash University, Prahran, Melbourne, VIC 3181, Australia

*These two authors contributed equally to this work.

*^Corresponding author: Kalifa Bojang, Disease Control and Elimination Theme, Medical Research Council Unit, Atlantic Road, Fajara, P.O. Box 273, Banjul, The Gambia. E-mail:* [*kbojang@mrc.gm*](mailto:kbojang@mrc.gm)*. Telephone: +220-9961094*

**Running title: Malaria vectored vaccines in African children**

**Supplementary Tables**

**Table S1**. Local solicited adverse events during 3 day follow up after ChAd63 vaccination (or first dose HDRCV where relevant). NA=not assessed.

|  | **Group** | | | | | | | | | | | | |
| --- | --- | --- | --- | --- | --- | --- | --- | --- | --- | --- | --- | --- | --- |
| **n (%)** | **1a** | **1b** | **1c** | **1d** | **1e** | **1f** | **2a** | **2b** | **2c** | **3a** | **3b** | **3c** | **4** |
| **Pain at injection site / Limitation of arm movement** | 0 | 2 (40.0) | 1 (16.7) | 4 (66.7) | 1 (16.7) | 0 | 0 | 0 | 0 | 0 | 0 | 0 | 5 (16.7) |
| **Redness/ discolouration at injection site** | 0 | 0 | 0 | 0 | 0 | 0 | 1  (8.3) | 0 | 0 | 1  (8.3) | 0 | 0 | 0 |
| **Swelling at injection site** | 0 | 0 | 0 | 0 | 0 | 0 | 0 | 1 (8.3) | 0 | 2 (16.7) | 1  (8.3) | 0 | 0 |
| **Warmth at injection site** | 0 | 0 | 0 | 0 | 0 | 0 | 0 | 0 | 0 | 0 | 0 | 0 | NA |
| **Scaling/pustule/blistering at injection site** | 0 | 0 | 0 | 0 | 0 | 0 | 0 | 0 | 0 | 1 (8.3) | 0 | 0 | NA |

**Table S2. Systemic solicited adverse events during 3 day follow up after ChAd63 vaccination (or first dose HDRCV where relevant) NA=not assessed**

|  | **Group** | | | | | | | | | | | | |
| --- | --- | --- | --- | --- | --- | --- | --- | --- | --- | --- | --- | --- | --- |
| **n (%)** | **1a** | **1b** | **1c** | **1d** | **1e** | **1f** | **2a** | **2b** | **2c** | **3a** | **3b** | **3c** | **4** |
| **Documented fever (>37.5)** | 1(20.0) | 0 | 0 | 1(16.7) | 0 | 1(16.7) | 2(16.7) | 2(16.7) | 2(16.7) | 0 | 1(8.3) | 1(8.3) | 4(13.3) |
| **Fever reported by carer** | 1(20.0) | 0 | 0 | 0 | 4(66.7) | 1(16.7) | 3(25.0) | 6(50.0) | 1(8.3) | 4 (33.3) | 6(50.0) | 2(16.7) | 15(50.0) |
| **Reduced oral intake/ Refusal to feed** | 0 | 0 | 0 | 0 | 0 | 0 | 0 | 0 | 0 | 0 | 0 | 0 | 3(10.0) |
| **Reduced activity** | 0 | 0 | 0 | 0 | 0 | 0 | 0 | 0 | 0 | 0 | 0 | 0 | NA |
| **Irritability** | 0 | 0 | 0 | 0 | 0 | 0 | 0 | 0 | 0 | 0 | 0 | 0 | 1 (3.3) |
| **Drowsiness** | 0 | 0 | 0 | 0 | 0 | 0 | 0 | 0 | 0 | 0 | 0 | 0 | 13(43.3) |
| **Vomiting** | 0 | 0 | 0 | 0 | 0 | 0 | 0 | 1 (8.3) | 0 | 0 | 0 | 0 | NA |
| **Diarrhoea** | 0 | 1  (20.0) | 0 | 0 | 0 | 0 | 2(16.7) | 0 | 1(8.3) | 2 (16.7) | 0 | 1(8.3) | NA |
| **Excessive crying** | 0 | 0 | 0 | 0 | 0 | 0 | 0 | 0 | 0 | 0 | 0 | 0 | NA |
| **Others:** |  |  |  |  |  |  |  |  |  |  |  |  | 0 |
| **Skin rash** | 0 | 0 | 0 | 1(16.7) | 0 | 0 | 0 | 0 | 0 | 0 | 1(8.3) | 1(8.3) |  |
| **Scalp abscess** | 0 | 0 | 0 | 0 | 0 | 0 | 0 | 0 | 0 | 1 (8.3) | 0 | 0 |  |
| **Facial swelling following fall** | 0 | 0 | 0 | 0 | 0 | 0 | 0 | 0 | 1(8.3) | 0 | 0 | 0 |  |
| **Facial boil** | 0 | 0 | 0 | 0 | 0 | 0 | 0 | 0 | 1(8.3) | 0 | 0 | 0 |  |
| **Chicken pox** | 1(20.0) | 0 | 0 | 0 | 0 | 0 | 0 | 0 | 0 | 0 | 0 | 0 |  |
| **Cough** | 0 | 2  (40.0) | 1  (16.7) | 0 | 0 | 0 | 0 | 0 | 0 | 0 | 0 | 0 |  |
| **Tinea capitis** | 0 | 0 | 0 | 0 | 0 | 1  (16.7) | 0 | 0 | 0 | 0 | 0 | 0 |  |
| **Ulcer on the forehead** | 0 | 0 | 0 | 0 | 1  (16.7) | 0 | 0 | 0 | 0 | 0 | 0 | 0 |  |

|  | **Group** | | | | | | | | | | | | |
| --- | --- | --- | --- | --- | --- | --- | --- | --- | --- | --- | --- | --- | --- |
| **n (%)** | **1a** | **1b** | **1c** | **1d** | **1e** | **1f** | **2a** | **2b** | **2c** | **3a** | **3b** | **3c** | **4** |
| **Pain at injection site/limitation of arm movement** | 1  (20.0) | 0 | 0 | 0 | 1 (16.7) | 0 | 0 | 0 | 0 | 0 | 0 | 0 | 13 (43.3) |
| **Redness at injection site** | 0 | 0 | 0 | 0 | 0 | 0 | 0 | 0 | 0 | 0 | 0 | 0 | 1 (3.3) |
| **Swelling at injection site** | 0 | 0 | 0 | 0 | 0 | 0 | 1 (8.3) | 0 | 0 | 0 | 0 | 0 | 0 |
| **Warmth at injection site** | 0 | 0 | 0 | 0 | 0 | 0 | 0 | 0 | 0 | 0 | 0 | 0 | NA |
| **Scar at injection site** | 0 | 0 | 0 | 1 (16.7) | 0 | 0 | 0 | 0 | 0 | 0 | 0 | 0 | NA |

**Table S3. Local solicited adverse events during 3 day follow up after MVA vaccination (or second dose HDRCV where relevant). NA=not assessed.**

**Table S4. Systemic solicited adverse events during 3 day follow up after MVA vaccination (or second dose HDRCV where relevant) NA=not assessed.**

|  | **Group** | | | | | | | | | | | | |
| --- | --- | --- | --- | --- | --- | --- | --- | --- | --- | --- | --- | --- | --- |
| **n (%)** | **1a** | **1b** | **1c** | **1d** | **1e** | **1f** | **2a** | **2b** | **2c** | **3a** | **3b** | **3c** | **4** |
| **Documented fever (>37.5°C)** | 0 | 1 (20.0) | 1(16.7) | 0 | 0 | 0 | 2(16.7) | 0 | 0 | 0 | 0 | 0 | 9 (30) |
| **Fever reported by carer** | 0 | 1 (20.0) | 0 | 2(33.3) | 0 | 0 | 0 | 5(41.7) | 0 | 2(16.7) | 3(25.0) | 1(8.3) | 13(43.3) |
| **Reduced oral intake/ Refusal to feed** | 0 | 0 | 1(16.7) | 0 | 0 | 0 | 0 | 0 | 1(8.3) | 1(8.3) | 0 | 0 | 4 (13.3) |
| **Reduced activity** | 1 (20.0) | 0 | 0 | 1(16.7) | 1(16.7) | 0 | 0 | 0 | 0 | 0 | 0 | 0 | NA |
| **Irritability** | 0 | 0 | 0 | 0 | 0 | 0 | 0 | 0 | 0 | 0 | 0 | 0 | 1 (3.3) |
| **Drowsiness** | 0 | 0 | 0 | 0 | 0 | 0 | 0 | 0 | 0 | 0 | 0 | 0 | 3 (10) |
| **Vomiting** | 0 | 0 | 0 | 0 | 0 | 0 | 0 | 2(16.7) | 1(8.3) | 0 | 0 | 0 | NA |
| **Diarrhoea** | 0 | 0 | 0 | 0 | 1(16.7) | 0 | 0 | 1(8.3) | 2(16.7) | 1(8.3) | 0 | 0 | NA |
| **Excessive crying** | 0 | 0 | 0 | 0 | 0 | 0 | 0 | 0 | 0 | 0 | 0 | 0 | NA |
| **Others:** |  |  |  |  |  |  |  |  |  |  |  |  | 0 |
| **Cough** | 1(20.0) | 0 | 0 | 0 | 0 | 0 | 2(16.7) | 0 | 0 | 0 | 0 | 0 |  |
| **Boil on the scalp** | 0 | 0 | 0 | 0 | 0 | 0 | 0 | 1 (8.3) | 0 | 0 | 0 | 0 |  |
| **Watery nasal discharge** | 0 | 1 (20.0) | 0 | 0 | 0 | 0 | 1(8.3) | 0 | 0 | 0 | 0 | 0 |  |
| **Jaw swelling** | 0 | 0 | 0 | 1(16.7) | 0 | 0 | 0 | 0 | 0 | 0 | 0 | 0 |  |
| **Skin rash** | 0 | 0 | 0 | 0 | 0 | 1(16.7) | 0 | 0 | 0 | 0 | 0 | 0 |  |
| **Persistent elevated ALT** | 0 | 0 | 0 | 0 | 1(16.7) | 0 | 0 | 0 | 0 | 0 | 0 | 0 |  |
| **Watery nasal discharge** | 0 | 1(20.0) | 0 | 0 | 0 | 0 | 0 | 0 | 0 | 0 | 0 | 0 |  |
| **Chicken pox** | 0 | 0 | 0 | 1(16.7) | 0 | 0 | 0 | 0 | 0 | 0 | 0 | 0 |  |
| **Angular stomatitis** | 0 | 1(20.0) | 0 | 0 | 0 | 0 | 0 | 0 | 0 | 0 | 0 | 0 |  |

**Table S5. Incidence of unsolicited AEs in 28 days post immunisation with ChAd63 ME-TRAP (NA=not assessed)**

|  | **Group** | | | | | | | | | | | | |
| --- | --- | --- | --- | --- | --- | --- | --- | --- | --- | --- | --- | --- | --- |
| **n (%)** | **1a** | **1b** | **1c** | **1d** | **1e** | **1f** | **2a** | **2b** | **2c** | **3a** | **3b** | **3c** | **4** |
| **Fever reported by carer** | 0 | 0 | 0 | 2(33.3) | 3(50.0) | 1(16.7) | 0 | 1 (8.3) | 0 | 0 | 0 | 0 | 0 |
| **Fever** | 1(20.0) | 0 | 0 | 0 | 0 | 0 | 0 | 1 (8.3) | 1 (8.3) | 1 (8.3) | 0 | 0 | NA |
| **Diarrhoea** | 0 | 0 | 0 | 0 | 0 | 0 | 0 | 1 (8.3) | 0 | 0 | 0 | 0 | 4(13.3) |
| **Rhinitis** | 0 | 0 | 0 | 0 | 0 | 0 | 1(8.3) | 0 | 0 | 0 | 0 | 0 | 6 (20) |
| **Vomiting** | 0 | 0 | 0 | 0 | 0 | 0 | 0 | 1 (8.3) | 0 | 0 | 0 | 0 | 0 |
| **Urinary tract infection** | 0 | 0 | 0 | 0 | 0 | 0 | 0 | 0 | 0 | 0 | 0 | 0 | 1 (3.3) |
| **Local swelling** | 0 | 0 | 0 | 1(16.7) | 0 | 0 | 1(8.3) | 0 | 0 | 0 | 0 | 0 | 0 |
| **Elevated ALT** | 0 | 0 | 0 | 0 | 0 | 0 | 0 | 1 (8.3) | 0 | 0 | 0 | 0 | 0 |
| **Acute respiratory infection** | 0 | 0 | 0 | 1(16.7) | 0 | 0 | 0 | 0 | 0 | 0 | 0 | 0 | 12 (40) |
| **Decreased oral intake** | 1(20.0) | 0 | 0 | 0 | 0 | 0 | 0 | 0 | 0 | 0 | 0 | 0 | 0 |
| **Conjunctivitis** | 0 | 0 | 0 | 0 | 0 | 0 | 0 | 0 | 0 | 0 | 0 | 0 | 3 (10) |
| **Worms** | 0 | 0 | 0 | 0 | 0 | 0 | 0 | 0 | 0 | 0 | 0 | 0 | 2 (6.7) |

**Table S6. Incidence of unsolicited AEs in 28 days post immunisation with MVA ME-TRAP (NA=not assessed)**

|  | **Group** | | | | | | | | | | | | |
| --- | --- | --- | --- | --- | --- | --- | --- | --- | --- | --- | --- | --- | --- |
| **n (%)** | **1a** | **1b** | **1c** | **1d** | **1e** | **1f** | **2a** | **2b** | **2c** | **3a** | **3b** | **3c** | **4** |
| **Fever reported by carer** | 0 | 1(20.0) | 0 | 0 | 0 | 0 | 0 | 0 | 0 | 0 | 0 | 0 | 0 |
| **Drop in Hb** | 0 | 0 | 0 | 0 | 0 | 0 | 0 | 1 (8.3) | 0 | 0 | 0 | 0 | 0 |
| **Cough** | 0 | 0 | 0 | 1(16.7) | 0 | 0 | 0 | 1 (8.3) | 0 | 0 | 0 | 0 | 0 |
| **Oral thrush** | 0 | 0 | 0 | 0 | 0 | 0 | 0 | 1 (8.3) | 0 | 0 | 0 | 0 | 0 |
| **Diarrhoea** | 0 | 0 | 0 | 0 | 0 | 0 | 0 | 0 | 0 | 0 | 0 | 0 | 2 (6.7) |
| **Chicken pox** | 0 | 1(20.0) | 0 | 0 | 0 | 0 | 0 | 0 | 0 | 0 | 0 | 0 | 0 |
| **Limitation of arm movement** | 0 | 1(20.0) | 0 | 0 | 0 | 0 | 0 | 0 | 0 | 0 | 0 | 0 | 0 |
| **Acute respiratory infection** | 0 | 0 | 0 | 0 | 0 | 0 | 0 | 0 | 0 | 0 | 0 | 0 | 8 (26.7) |
| **Gingivo-stomatitis** | 0 | 0 | 0 | 0 | 0 | 0 | 0 | 0 | 0 | 0 | 0 | 0 | 1 (3.3) |
| **Tonsilitis** | 0 | 0 | 0 | 0 | 0 | 0 | 0 | 0 | 0 | 0 | 0 | 0 | 1 (3.3) |
